# Structured beam-driven multipolar mode control in nanoparticles

**DOI:** 10.1515/nanoph-2025-0465

**Published:** 2025-11-20

**Authors:** Asma Fallah, Eileen Otte

**Affiliations:** The Institute of Optics, 6927 University of Rochester, Rochester, NY 14627, USA

**Keywords:** structured light, nanoparticle scattering, generalized cylindrical vector beam, polarization control, generalized Lorenz–Mie theory, light–matter interaction

## Abstract

Due to their unique tight focusing properties, structured light beams, such as cylindrical vector beams, offer unique opportunities for tailoring light–matter interaction at the nanoscale. In this work, we investigate the scattering response of a spherical nanoparticle illuminated by a Focused Generalized Cylindrical Vector Beam (FGCVB). We employ a full vectorial framework – numerically and analytically. We model the focal field distribution of the FGCVB, compute and examine the scattered fields using generalized Lorenz–Mie theory, and analyze the influence of beam polarization structure on the scattering cross section and multipole content of the scattered fields. We find that tailoring the polarization composition of the incident FGCVB allows selective excitation of and tuning between electric and magnetic dipolar as well as quadrupolar modes, which offers a pathway for polarization-controlled light scattering at the nanoscale. We also examine and employ the influence of focal point position and numerical aperture of the lens on the scattered field. This work expands our understanding of vector beam scattering and provides design principles for polarization-resolved nano-optical spectroscopy and microscopy.

## 1 Introduction

Controlling light–matter interactions, while also enabling new functionalities, lies at the core of nanophotonics; it is crucial for applications such as ultra-sensitive sensors  [1], quantum technologies  [2], [3], high-resolution imaging  [4], [5], and energy harvesting [6], [7]. Conventionally, this control is achieved by nanostructuring the materials involved in the interaction, and thereby, shaping the flow of light [8], [9]. In a complementary approach, controlling the incident light field of the interaction has gained significant attention. For instance, it has been shown that so-called structured light fields [10], [11], [12] – fields that are spatially varying in amplitude, phase, polarization, or other properties – allow us to multiplex functionalities into nanostructured metasurfaces [13] for applications such as advanced imaging or quantum cryptography [14], [15], [16]. Beyond this, structured light opens new possibilities for controlling interactions at even smaller scales, namely, interactions of light with single nanoscale objects [17], [18].

Considering the interaction of a single nanoparticle with structured light is particularly intriguing due to the unique focusing properties of these light fields. Polarization-structured, i.e., vectorial light has the ability to generate strong longitudinal (*E*_*z*_) in additional to typical transverse (*E*_*x*,*y*_) electric field components when tightly focused through high numerical aperture (NA) optics [19]. This three-dimensional (3D) nature of the electromagnetic field in light’s focus provides additional degrees of freedom to tailor and even unlock usually inaccessible interactions at the nanoscale.

Among the best-known examples of vectorial beams are cylindrical vector beams (CVBs) [19], [20], [21] of spatially varying polarization. Their focusing properties have proven beneficial for various applications, including high-resolution microscopy  [22], [23], optical trapping and particle manipulation [24], [25], and laser machining [26], [27], [28], [29], [30]. A natural extension of CVBs is the so-called generalized cylindrical vector beam (GCVB), which is a linear superposition of radial and azimuthal polarization components with an adjustable mixing angle that controls the relative amplitude and phase between these components. Such beams enable precise shaping of the focal field distribution per polarization composition (*E*_*x*,*y*,*z*_) [31], [32], since focusing purely radial or azimuthal vector beams represent the two extreme cases of maximum or minimum (i.e., no) longitudinal polarization contributions (*E*_*z*_) in the focus, respectively. This beam shaping capability has proven particularly useful for tailoring the excitation conditions in nanoscale scattering experiments [30], [33], [34], [35].

The scattering of spherical nanoparticles under plane wave or Gaussian beam illumination has been extensively characterized  [36], [37]. However, despite the growing interest and potential of structured light scattering, systematic studies investigating how GCVBs modify the multipolar scattering response remain limited. While tightly focused GCVBs have been shown to selectively excite specific multipolar resonances in dielectric nanoparticles, enabling control over electric versus magnetic dipole contributions and higher-order multipoles such as quadrupoles [33], GCVBs offer additional tunability through their polarization mixing capabilities, focal positioning, and the numerical aperture of the focusing optics [38]. These combined parameters present new opportunities for on-demand scattering control that remain underexplored.

In this work, by combining the Richards–Wolf formalism with generalized Lorenz–Mie theory, we obtain a detailed picture of how the incident beam structure influences, hence, can be used to control the excitation of resonant modes in spherical dielectric nanoparticles. Besides our analytical analysis, we numerically investigate the scattering of a spherical nanoparticle by a tightly focused GCVB (FGCVB) and compare both. We analyze how the polarization of the incident beam influences the excitation of different multipolar modes and the resulting scattering cross sections, while also examining the effects of the numerical aperture of the lens and the focal point position relative to the scatterer. Our analytical and numerical analyses reveal that by tailoring the radial–azimuthal mixture, one can selectively and smoothly enhance or suppress electric and magnetic dipole contributions, as well as quadrupole responses, providing a powerful mechanism for adjustable, polarization-controlled light–matter interactions at the nanoscale. Distinct from previous experimental or application-driven studies of vectorial beam–particle interactions, our work introduces a unified full-vectorial analytical–numerical framework that couples the Richards–Wolf focusing model with generalized Lorenz–Mie theory, enabling quantitative decomposition of electric and magnetic multipoles under arbitrary polarization mixing and focusing conditions.

## 2 Results

### 2.1 Theoretical framework

Cylindrical vector beams (CVBs) are characterized by spatially varying polarization states that possess cylindrical symmetry [19], [20]. Conventionally, these beams can be expressed as a superposition of two orthogonally polarized, helical Laguerre–Gaussian beams (LG_*p*,*ℓ*_, *p*, *ℓ*: radial, azimuthal mode number), namely.

(1) $${\vec{E}}_{\text{CVB}}\propto {\vec{e}}_{R}\cdot {\text{LG}}_{p,\ell }\cdot {\text{e}}^{\text{i}\alpha /2}+{\vec{e}}_{L}\cdot {\text{LG}}_{p,-\ell }\cdot {\text{e}}^{-\text{i}\alpha /2}.$$

**Figure 1:**
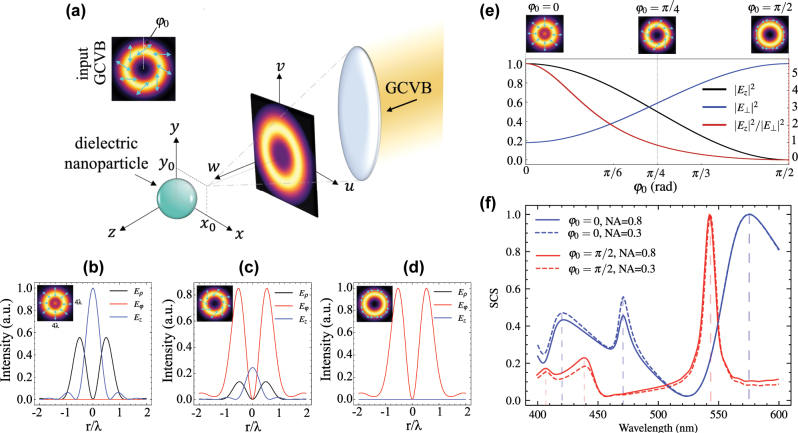
The approach for structured beam-driven multipolar mode control. (a) Conceptual schematic of the approach, introducing parameters of the theoretical framework (top left: incident generalized cylindrical vector beam (GCVB)). (b–d) Electric field components at the focal plane (*z* = 0 and *ρ*_0_ = 0) in the absence of the nanosphere for three polarization rotation angles: (b) *φ*_0_ = 0, (c) *φ*_0_ = *π*/4, and (d) *φ*_0_ = *π*/2. The insets in panels (b–e) sketch the intensity distributions in the transverse plane (4*λ* × 4*λ*), with blue arrows indicating the transverse polarization. (e) Normalized longitudinal and transverse intensity components of the electric field (black and blue line; left vertical axis), along with their ratio (red line; right, red vertical axis), as a function of the polarization angle at the focal point (*r* = 0) (normalization factor is the maximum of total electric field at the focal point). Insets above the graph visualize the focal field intensity and vectors of selected examples (cf. (b–d)) and their location in the graph. (f) Normalized scattering cross section (SCS) for two different sets of beam parameters with *ρ*_0_ = 50 nm and *ϕ*_0_ = *π*/2 for the incident beam (normalization factor is the maximum of each case); dashed vertical lines (light blue and light red) mark resonances. Here, NA = 0.8 unless otherwise noted.

Here, ${\vec{e}}_{R,L}$ correspond to the unit vectors of right and left circular polarization in Jones formalism. These typical CVBs include radially and azimuthally polarized beams (*ℓ* = ±1, *p* = 0, *α* = {0, *π*}), with generalized forms incorporating a tunable polarization rotation angle *φ*_0_ that is the rotation angle from the radial axis that allows continuous variation between these states (Figure 1(a), top left). When focused through a high numerical aperture (NA) objective, conventional CVBs and generalized CVBs (GCVBs) can exhibit strong longitudinal electric field components – formed by initially radial oriented input components – enabling the customization of focal field profiles of *E*_*x*,*y*,*z*_  [12], [31]. To rigorously model these focal field distributions, we employ the Richards–Wolf vectorial diffraction formalism, which accounts for strong longitudinal electric field components in the nonparaxial regime of light  [39], [40].

The focal field of a focused GCVB is a superposition of radially, azimuthally, and longitudinally polarized components, i.e.,

(2) $$\vec{E}\left(\rho ,\varphi ,z\right)=E_{\rho }\;{\vec{e}}_{\rho }+E_{\varphi }\;{\vec{e}}_{\varphi }+E_{z}\;{\vec{e}}_{z},$$

where the *E*_*r*_, *E*_*φ*_, and *E*_*z*_ can be derived in *O*_*xyz*_ coordinates using the Richards–Wolf formalism; the electric field near the focus of a high-NA lens is [19], [31]:

(3) $$\begin{array}{ll} E_{\rho }\left(\rho ,\varphi ,z\right) & =A\cos\varphi_{0}\overset{\theta_{\max}^{\prime }}{\underset{0}{\int }}\cos^{1/2}\left(\theta^{\prime }\right)P\left(\theta^{\prime }\right)\sin\theta^{\prime }\cos\theta^{\prime }\\ & \;\times J_{1}\left(k\rho^{\prime }\sin\theta^{\prime }\right){\text{e}}^{\text{i}kz\cos\theta^{\prime }}\;d\theta^{\prime }, \end{array}$$

(4) $$\begin{array}{ll} E_{\varphi }\left(\rho ,\varphi ,z\right) & =A\sin\varphi_{0}\overset{\theta_{\max}^{\prime }}{\underset{0}{\int }}\cos^{1/2}\left(\theta^{\prime }\right)P\left(\theta^{\prime }\right)\\ & \;\times J_{1}\left(k\rho^{\prime }\sin\theta^{\prime }\right){\text{e}}^{\text{i}kz\cos\theta^{\prime }}\;d\theta^{\prime }, \end{array}$$

(5) $$\begin{array}{ll} E_{z}\left(\rho ,\varphi ,z\right) & =iA\cos\varphi_{0}\overset{\theta_{\max}^{\prime }}{\underset{0}{\int }}\cos^{1/2}\left(\theta^{\prime }\right)P\left(\theta^{\prime }\right)\sin^{2}\theta^{\prime }\\ & \;\times J_{0}\left(k\rho^{\prime }\sin\theta^{\prime }\right){\text{e}}^{\text{i}kz\cos\theta^{\prime }}\;d\theta^{\prime } \end{array}$$

with $\rho^{\prime }=\sqrt{\rho^{2}+\rho_{0}^{2}-2\rho \rho_{0}\cos\left(\phi -\phi_{0}\right)}$ . Here, $\theta_{\max}^{\prime }=\arcsin\left(\text{NA}/n_{\text{medium}}\right)$ is the maximal angle determined by the numerical aperture of the objective lens (NA) and assuming the particle is located in vacuum (*n*_medium_ = 1). *P*(*θ*′) is the pupil apodization function, and *A* is a normalization factor. Moreover, *k* and *J*_*n*_ are the wavenumber and the Bessel function of first kind with order *n*. Further, *ρ*_0_ and *ϕ*_0_ denote the location of the focal point in the *O*_*xyz*_ coordinate system, where $\rho_{0}=\sqrt{x_{0}^{2}+y_{0}^{2}}$ and *ϕ*_0_ = arctan(*y*_0_/*x*_0_).

In this study, as illustrated in Figure 1(a), we assume that a dielectric nanosphere of diameter *d* = 185 nm and relative permittivity of *ɛ*_*s*_ is illuminated by the focused GCVB; the sphere is crystalline silicon [41], located in a vacuum. Note that although these assumptions are made here, the theoretical framework is not limited to them and can be easily extended or modified (see Section 3).

In Figure 1(b)–(d), we exemplify the focal field distributions by three cases of tightly focusing a (b) purely radial (*φ*_0_ = 0), (c) *φ*_0_ = *π*/4, and (d) azimuthal (*φ*_0_ = *π*/2) GCVB (NA = 0.8). Note that for (c) *φ*_0_ = *π*/4, the spiral-type GCVB has a 1:1 ratio of radial and azimuthal polarization components, exemplifying the effect of tuning the polarization angle of the GCVB. For each example, we depict the corresponding intensity profiles of transverse and longitudinal electric field components *E*_*ρ*,*φ*_ and *E*_*z*_, respectively, at the focal plane (*z* = 0). As insets, we present the respective total focal field intensity in the *xoy*-plane; blue arrows indicate the transverse polarization distribution. It is evident that the longitudinal component is dominant for the radially polarized input GCVB (b), whereas it vanishes for *φ*_0_ = *π*/2, corresponding to an azimuthally polarized input GCVB (d). Adjusting the polarization angle *φ*_0_ can be seen as tuning between the two presented extreme cases of focused (b) radial and (d) azimuthal GCVB as confirmed by Figure 1(c). Figure 1(e) further highlights this *φ*_0_-dependent tunability, presenting the contributions of transverse to longitudinal polarization components in the field focus for varying the ratio of radial to azimuthal components in the back focal plane (BFP) of the focusing objective (NA = 0.8; left axis: |*E*_*z*,⊥_|^2^; right axis: |*E*_*z*_|^2^/|*E*_⊥_|^2^).

For the theoretical analysis of the field scattered by the nanosphere, when illuminated by these structured focal fields, we express the incident field as a series expansion in spherical multipole basis functions such that generalized Lorenz–Mie theory can be used to study the scattering. Here, to derive the expansion coefficients, which are called the beam shape coefficients, we employ the Bromwich formulation [42]. This approach allows us to express the electromagnetic fields in terms of two canonical solutions – transverse magnetic (TM) and transverse electric (TE) modes – while ensuring that the boundary conditions imposed by Maxwell’s equations are satisfied. To compute the beam shape coefficients, we adopt the integral localized approximation of the incident field and evaluate them using the following expressions [42], [43]:

(6) $$g_{n,TE}^{m}=\frac{Z_{n}^{m}}{2\pi H_{0}}\overset{2\pi }{\underset{0}{\int }}H_{r,\text{loc}}\left(r,\theta ,\phi \right){\text{e}}^{-\text{i}m\phi }\;d\phi ,$$

(7) $$g_{n,TM}^{m}=\frac{Z_{n}^{m}}{2\pi E_{0}}\overset{2\pi }{\underset{0}{\int }}E_{r,\text{loc}}\left(r,\theta ,\phi \right){\text{e}}^{-\text{i}m\phi }\;d\phi ,$$

where

(8) $$Z_{n}^{m}=\left\{\begin{array}{cc} \frac{2n\left(n+1\right)\text{i}}{2n+1},\; & m=0,\\ {\left(\frac{-2\text{i}}{2n+1}\right)}^{|m|-1},\; & m\neq 0. \end{array}\right.$$

Once the beam shape coefficients $g_{n}^{m}$ are determined, they serve as weighting factors that project the incident structured field onto the spherical multipole basis. Here, *n* and *m* denote the multipole order and azimuthal index, respectively, corresponding to the degree and order of the associated spherical harmonics that describe the angular dependence of the scattered fields. *E*_0_ and *H*_0_ represent the reference amplitudes of the incident electric and magnetic fields, which serve as normalization constants in the beam–shape–coefficient definitions. The total scattered field is going to be obtained as a linear combination of these multipole contributions, each weighted by its respective $g_{n}^{m}$ and the corresponding Lorenz–Mie scattering coefficients (see Supplementary for details). From the scattered field, one can then compute physically relevant quantities such as the near-field distribution, the scattering amplitude, or the scattering cross section.

*H*_*r*,loc_ and *E*_*r*,loc_ represent the localized radial components of the incident beam, which can be easily derived after transferring the beam into spherical coordinates (see Supplementary for details). The localized radial component of electric and magnetic field can be derived as following:

(9) $$\begin{array}{ll} E_{r,\text{loc}}\left(\rho ,\varphi ,z\right) & =A\cos\varphi_{0}\overset{\theta_{\max}^{\prime }}{\underset{0}{\int }}\cos^{3/2}\left(\theta^{\prime }\right)P\left(\theta^{\prime }\right)\sin\theta^{\prime }\\ & \;\times J_{1}\left(\zeta^{1/2}\sin\theta^{\prime }\right)\;d\theta^{\prime }, \end{array}$$

(10) $$\begin{array}{ll} H_{r,\text{loc}} & =\frac{A\cos\varphi_{0}k^{2}\rho_{0}\sin\left(\phi -\phi_{0}\right)}{\omega \zeta^{1/2}}\overset{\theta_{\max}^{\prime }}{\underset{0}{\int }}\cos^{1/2}\left(\theta^{\prime }\right)P\left(\theta^{\prime }\right)\\ & \;\times \sin^{3}\theta^{\prime }J_{1}\left(\zeta^{1/2}\sin\theta^{\prime }\right)\;d\theta^{\prime }\\ + & \;\frac{Ak}{\omega }\sin\varphi_{0}\overset{\theta_{\max}^{\prime }}{\underset{0}{\int }}\cos^{3/2}\left(\theta^{\prime }\right)P\left(\theta^{\prime }\right)J_{1}\left(\zeta^{1/2}\sin\theta^{\prime }\right)\;d\theta^{\prime } \end{array}$$

with $\zeta ={\left(n+\frac{1}{2}\right)}^{2}+\rho_{0}^{2}k^{2}-2\left(n+\frac{1}{2}\right)k\rho_{0}\cos\left(\phi -\phi_{0}\right)$ .

It is worth highlighting that the radial component *H*_*r*,loc_ in spherical coordinates is derived by utilizing the Maxwell relation $H_{\rho }=\frac{1}{-i\omega }\left(\frac{1}{\rho }\partial E_{z}/\partial \phi -\partial E_{\phi }/\partial z\right)$ and the axial component *H*_*z*_ (*H*_*r*_ = *H*_*ρ*_ sin *θ* + *H*_*z*_ cos *θ*; see Supplementary for details), which means that the longitudinal component of the electric field (*E*_*z*_) plays an important role in determining the *H*_*r*,loc_ value (first term in Eq. (10)). Crucially, choosing an on-axis beam configuration will eliminate the influence of *E*_*z*_ on *H*_*r*,loc_; to observe the effect of this component, we must consider off-axis beam configurations. Notably, minimal off-axis illumination more accurately reflects typical experimental conditions, where ideal alignment is rarely achieved. In the following, if not noted differently, we assume an off-axis illumination with *ρ*_0_ = 50 nm and *ϕ*_0_ = *π*/2.

### 2.2 Scattering cross section and modal decomposition

The scattering cross section (SCS) of the nanoparticle provides a direct measure of how strongly a nanoparticle interacts with the FGCVB, which can be derived using Generalized Lorenz–Mie Theory (GLMT) with Debye series expansion [44], [45], incorporating the calculated beam shape coefficients (for details, see Supplementary). Figure 1(f) depicts the normalized SCS of the FGCVB scattered by the spherical nanoparticle across the wavelength spectrum. In this work, the SCS is computed following the generalized Lorenz–Mie formalism, where the incident power is obtained by integrating the total Poynting flux. For comparison across different focusing conditions, all SCS spectra shown in Figure 1(f) are normalized to the maximum value of each individual case (for comparison, the absolute (non-normalized) SCS spectra are provided in the Supplementary Material). In this figure, the effects of polarization and numerical aperture on the normalized SCS spectral behavior at different wavelengths are demonstrated. Evidently, the beam polarization has a significant influence on the SCS with the red/blue curves corresponding to focused radial/azimuthal GCVB. Moreover, the numerical aperture also influences the SCS as it modifies both the strength of the longitudinal field component and the focal field distribution. A higher numerical aperture leads to stronger field confinement and steeper field gradients, which can enhance higher-order multipole contributions to the scattering process. However, for subwavelength particles, these contributions remain relatively minor, and the primary impact of the numerical aperture in our case is observed in the overall strength of the scattering cross section (see Supplementary Information). It is worth noting that in intermediate cases (*φ*_0_ = ]0, *π*/2[), the presence of a nonzero *z*-component at the place of the nanoparticle makes the SCS resemble that of the radially polarized case (*φ*_0_ = 0).

**Figure 2:**
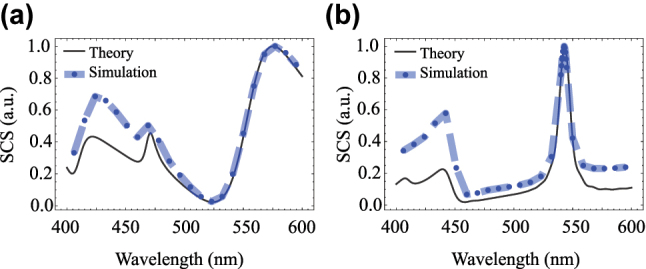
Comparison of the scattering cross section obtained from theoretical calculations and FDTD simulations for (a) a radially polarized incident beam (*φ*_0_ = 0) and (b) an azimuthally polarized beam (*φ*_0_ = *π*/2). The incident beam is focused through a lens with numerical aperture NA = 0.8, with *ρ*_0_ = 50 nm and *ϕ*_0_ = *π*/2.

To validate our results, Figure 2 compares the theoretically derived SCSs with FDTD simulations (Tidy3D, Flexcompute) for the two orthogonal extreme cases of a focused (a) radial and (b) azimuthal GCVB. It is important to note that the integral localized approximation [44], [46] employed for computing the beam shape coefficients shows wavelength-dependent accuracy in our system. In our study, the nanosphere with diameter *d* = 185 nm corresponds to size parameters ranging from *χ* ≈ 1.45 at *λ* = 400 nm to *χ* = *πd*/*λ* ≈ 0.96 at *λ* = 600 nm. Our results demonstrate that the approximation becomes more accurate at longer wavelengths (smaller size parameters), where excellent agreement is observed between theoretical predictions and FDTD simulations. The discrepancies at shorter wavelengths (larger size parameters within this range) suggest that for tightly focused cylindrical vector beams (the paraxial/localized assumption breaks down), the localized approximation may not adequately account for the complex near-field interactions and the detailed structure of the focused beam’s electromagnetic field distribution. This wavelength-dependent behavior highlights the importance of validating theoretical approximations against full-wave simulations, particularly when dealing with structured light beams and nanoscale scatterers [43].

**Figure 3:**
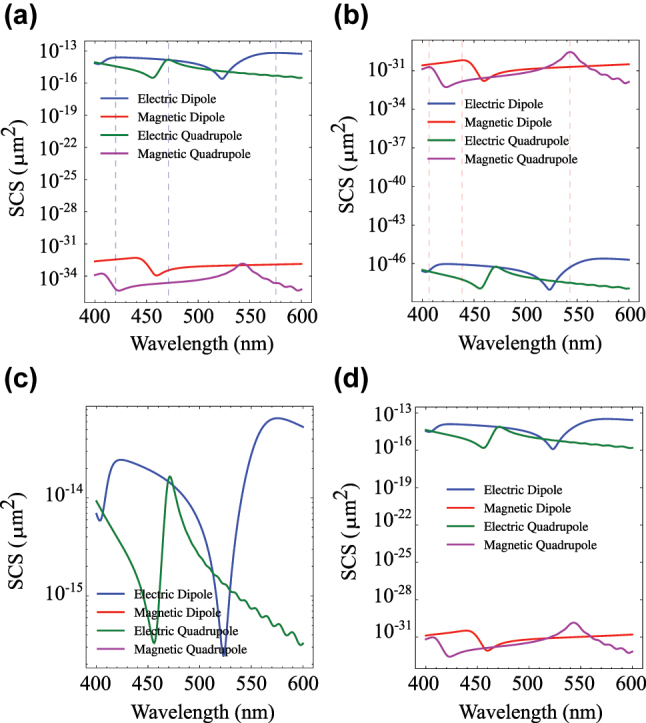
Mode decomposition for (a) *φ*_0_ = 0, (b) *φ*_0_ = *π*/2, (c) *φ*_0_ = 0, and (d) *φ*_0_ = *π*/4. Here, focusing NA = 0.8, *ρ*_0_ = 50 nm, and *ϕ*_0_ = *π*/2 for the incident beam in all panels except panel (c), which is *ρ*_0_ = 0. Dashed lines in panel (a–b) show the resonance frequencies marked in Figure 1(f).

To gain deeper insight into the structured-beam–particle interaction, we performed multipole mode decomposition. We calculate the scattered power from the Lorenz–Mie coefficients, quantifying the contribution of each multipolar mode (see Supplementary for details). This analysis enables a detailed understanding of how each multipole mode contributes to the overall scattering response. Figure 3(a) and (b) depicts the multipole mode decomposition for two polarization states of the incident beam, radially and azimuthally polarized, respectively. For the former case (a), electric modes are dominant, while magnetic modes are suppressed; in contrast, for the latter case (b), we observe the opposite, i.e., dominance of magnetic modes and suppression of electric modes.

The SCS for the focused radial GCVB (Figure 1(f), blue) revealed three resonances at about *λ* = 420 nm, *λ* = 470 nm, and *λ* = 575 nm; these locations are also marked by dashed lines in Figure 3(a). While for *λ* = 420 nm and *λ* = 575 nm, the dominant mode is clearly the electric dipole (blue curve), at *λ* = 470 nm, electric dipole and quadrupole (green curve) seem to be of equal contribution, thus coexisting. The SCS for the focused azimuthal GCVB (Figure 1(f), red), resonances are found at about *λ* = 405 nm, *λ* = 440 nm, and *λ* = 540 nm; again, these locations are marked by dashed lines in Figure 3(b). While for the resonances at shorter wavelength, the dominant mode is the magnetic dipole, at *λ* = 540 nm, we find the magnetic quadrupole being dominant.

Furthermore, Figure 3(c) presents the multipole mode decomposition of the radially polarized on-axis beam (*ρ*_0_ = 0). As discussed previously, when the focal point is exactly at the center of the particle, the effect of the longitudinal component of the electric field on *H*_*r*,loc_ is suppressed, and consequently no magnetic modes are excited.

Multipole mode decomposition of an intermediate case of (off-axis) FGCVB illumination is shown in Figure 3(d) for *φ*_0_ = *π*/4. It is evident that although electric modes remain dominant, the strength of the magnetic modes is enhanced compared to the radial polarization case of FGCVB. Thus, we observe the tunability of modal strength.

### 2.3 Field distributions

To understand the formation of these resonant modes under structured-light illumination and observations such as mode competition or coexistence, we examine the field distribution of the excited nanoparticle (Tidy3D, Flexcompute). This analysis also provides further insight into mode-tuning capabilities enabled by the adjustable parameters of FGCVBs.

**Figure 4:**
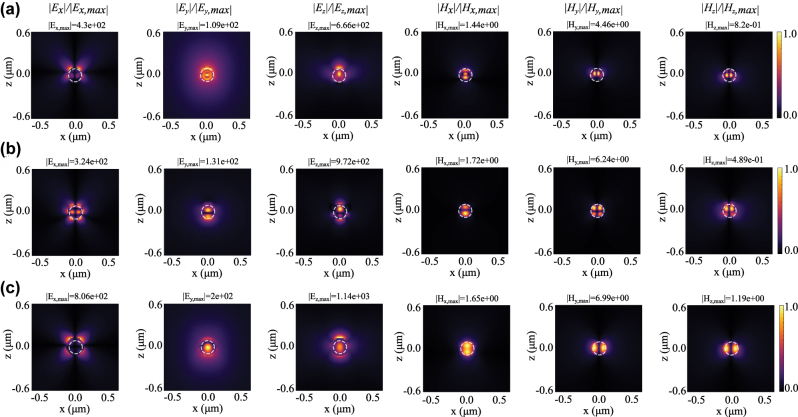
Normalized scattered field distributions of all components of electric and magnetic field of the excited nanosphere at *xoz* plane excited by radially polarized beam at (a) *λ* = 420 nm, (b) *λ* = 470 nm, and (c) *λ* = 575 nm. Here, the incident beam parameters are NA = 0.8, *ρ*_0_ = 50 nm, and *ϕ*_0_ = *π*/2. The normalization factor in each panel is the maximum field intensity, which is reported at the top of each figure, *E*_*x*,*y*,*z*, max_ (V/m) and *H*_*x*,*y*,*z*, max_ (A/m). Dashed white circles mark the boundary of the nanoparticle.

We analyze the field distribution of the excited nanoparticle for different beam parameters. Figure 4 shows the spatial distribution of all components of the scattered electric and magnetic field in the *xoz* plane at three representative wavelengths – corresponding to resonances observed in Figure 1(f). From left to right, we depict the normalized amplitudes of *E*_*x*,*y*,*z*_ and *H*_*x*,*y*,*z*_ for an off-axis beam with *φ*_0_ = 0 (i.e., radial) and numerical aperture (NA) of 0.8. The maximum field intensity is given above each penal. The location and size of the nanoparticle is indicated by a white dashed circle.

By focusing on the dominant field components per row, concentrated inside the particle, i.e., |*E*_*z*_| and |*H*_*y*_|, we observe a dipolar, overlapped dipolar and quadrupolar, and dipolar electric modal patterns at about (a) *λ* = 420 nm, (b) *λ* = 470 nm, and (c) *λ* = 575 nm, respectively. Also the magnetic fields clearly confirm these observations, which are overall in strong agreement with the theoretical mode decomposition presented in Figure 3(a).

**Figure 5:**
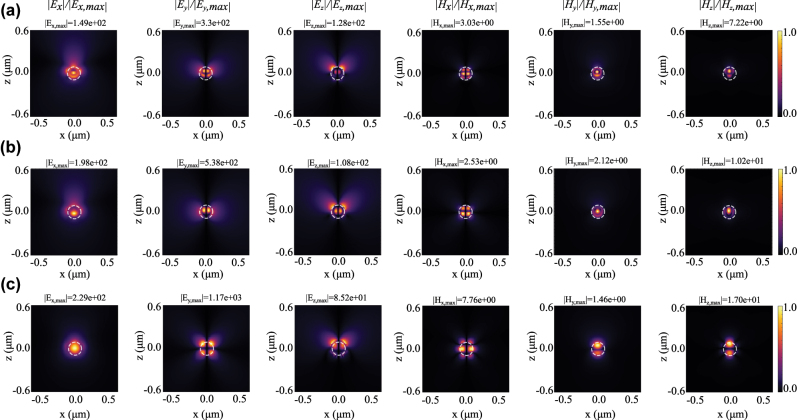
Normalized scattered field distributions of all components of the electric and magnetic fields of the excited nanosphere in the *xoz* plane when excited by an azimuthally polarized beam at (a) *λ* = 405 nm, (b) *λ* = 440 nm, and (c) *λ* = 540 nm. Here, the incident beam parameters are NA = 0.8, *ρ*_0_ = 50 nm, and *ϕ*_0_ = *π*/2. The normalization factor in each panel is the maximum field intensity, which is reported at the top of each figure, *E*_*x*,*y*,*z*, max_ (V/m) and *H*_*x*,*y*,*z*, max_ (A/m). Dashed white circles mark the boundary of the nanoparticle.

Similarly, assuming an off-axis azimuthal FGCVB as the incident beam (NA = 0.8), Figure 5 shows the spatial distribution of all components of the scattered electric and magnetic field at three representative wavelengths in the *xoz* plane. The selected wavelengths correspond to the resonances observed in Figure 1(f) with the mode decomposition presented in Figure 3(b). In Figure 5, we find dominant (a, b) magnetic dipolar and (c) quadrupolar modes while electric modes are relatively weak, confirming our modal decomposition (Figure 3(b)).

**Figure 6:**
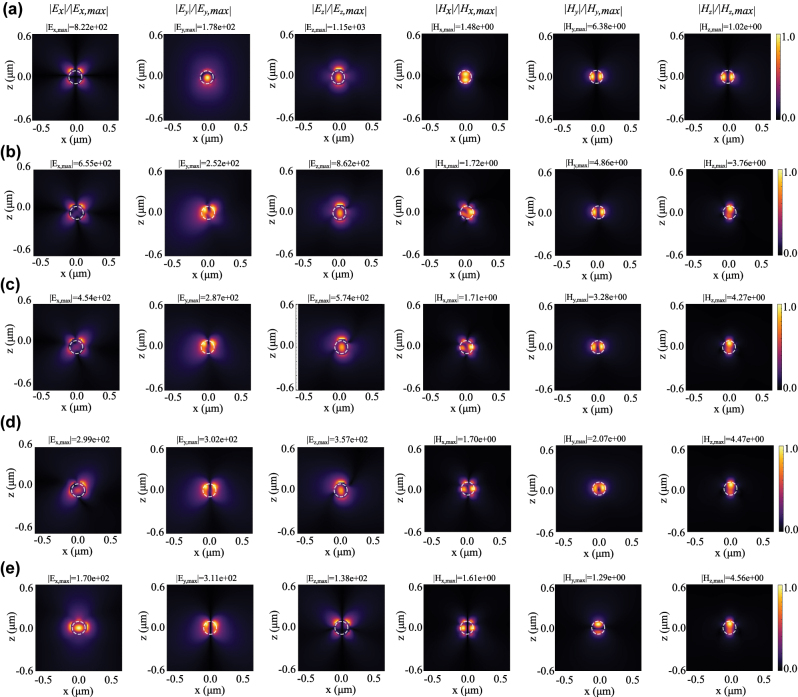
Normalized scattered field distributions of all components of electric and magnetic field of the excited nanosphere at *xoz* plane excited by (a) radially, (b) *φ*_0_ = *π*/6, (c) *π*/4, (d) *π*/3, and (e) azimuthally polarized beam at *λ* = 575 nm. Here, the incident beam parameters are NA = 0.8, *ρ*_0_ = 50 nm, and *ϕ*_0_ = *π*/2. The normalization factor in each panel is the maximum field intensity, which is reported at the top of each figure, *E*_*x*,*y*,*z*, max_ (V/m) and *H*_*x*,*y*,*z*, max_ (A/m). Dashed white circles mark the boundary of the nanoparticle.

Considering the field distribution of the FGCVBs without particle, some of the field distributions with/in the particle are to be expected (cf. Figure 1(b)–(d) and |*E*_*x*,*y*,*z*_|, |*H*_*x*,*y*,*z*_| in Figures 5 and 6). Clearly, the nanoparticle responds to the structured illumination, enabling selective excitation of electric and magnetic modes.

To investigate the flexibility of this selective approach further and understand the evolution between the two extreme cases of radial and azimuthal polarization, we determine the field distributions for the nanoparticle interacting with FGCVBs of various polarization rotation angles, namely *φ*_0_ = 0, *π*/6, *π*/4, *π*/3, *π*/2 (Figure 6(a)–(e), i.e., radial/top to azimuthal/bottom). The respective normalized scattered field components (|*E*_*x*,*y*,*z*_| and |*H*_*x*,*y*,*z*_|, left to right) are visualized in the Figure 6 for *λ* = 575 nm. Following the different components (per column) from top to bottom, one clearly observes how the dominant scattering mode transitions from electric dipole (for radial polarization) to magnetic dipole (for azimuthal polarization) by tuning the beam polarization. It can also be found that even small contributions of azimuthal field components to purely radial ones ((a)–(c)) cause a significant change in electric as well as magnetic field distributions at the particle, particularly visible for *E*_*y*_ and *H*_*z*_. Inversely, small contributions of radial field components to purely azimuthal ones ((e)–(d)) cause significant variations of the electromagnetic field, particularly visible in *E*_*z*_ and *H*_*y*_. This is explained by the presence and absence of longitudinal electric field components for a focused purely radial and azimuthal GCVB, respectively. For combinations of radial and azimuthal components, magnetic as well as electric dipolar and quadrupolar modes become dominant, with their contributions being tunable by the choice of polarization angle *φ*_0_ as shown in Figure 3(d).

## 3 Discussion

In conclusion, we investigated the tunable scattering response of dielectric nanoparticles illuminated by focused generalized cylindrical vector beams theoretically and numerically. Our results demonstrate that the polarization rotation angle provides precise control over multipolar mode excitation. The spatial field distributions confirm the selective excitation mechanism, revealing distinct multipolar patterns that correlate with theoretical analysis.

We present a complete theoretical framework, enabling the accurate prediction of multipolar mode excitation under structured beam illumination. Our framework is generalizable and not limited to the presented example of a dielectric particle or polarization rotation angle alone: particle size, shape, and material are adaptable parameters, as is the relative phase between radial and azimuthal incident polarization components. Further, since the Richards–Wolf focusing and the GLMT-based modal expansion enter our formulation independently, the Mie coefficients can be readily replaced by a T-matrix, enabling generalization to nonspherical or multilayered scatterers such as spheroids and core–shell particles, where the polarization parameter would similarly provide access to higher-order contributions. Also, while, in this work, we considered the nanoparticle in free space to isolate and analyze the fundamental role of structured beam polarization and focusing parameters, future work could additionally consider a substrate, which can significantly influence scattering spectra.

To match simulations to the experimental feasibility of our proposed structured beam-driven multipolar mode control, we consider parameters such as numerical aperture or *ρ*_0_ for common off-axis illumination. Future work could address further experimental imperfections such as material losses, complex geometries, and more. Given the availability of beam-shaping techniques based on, e.g., q-plates, spatial light modulators, digital mirror devices  [47], [48], [49], and modern integrated platforms [50], our proposed scheme is readily compatible with current experimental platforms.

While earlier reports have demonstrated polarization-dependent scattering experimentally, the novelty of this study lies especially in providing a rigorous, generalized theoretical foundation that bridges vectorial focusing and multipole decomposition within one consistent formalism, thereby clarifying and predicting selective mode excitation beyond previously empirical observations. As such, our work establishes a through framework for polarization-controlled light–matter interactions at the nanoscale, with direct applications in nano-optical spectroscopy, microscopy, and optical manipulation. The ability of our framework is exemplified by investigating the tuning between electric and magnetic responses through beam polarization, representing a significant advancement in structured light scattering control. Notably, tunable multipolar excitation provides opportunities for directional scattering in nanoantennas, the design of multifunctional metasurfaces, and the enhancement of nonlinear and quantum optical processes. The integration of the proposed theoretical framework with advanced beam-shaping technologies is expected to further expand the frontiers of tailored light–matter interactions at the nanoscale, encompassing the applications discussed above.

## Supplementary Material

Supplementary Material Details
