# Supplementary material for: Structured beam-driven multipolar mode control in nanoparticles
Source: Nanophotonics. 2025 Nov 20;14(24):4387–96. doi: 10.1515/nanoph-2025-0465 (PMC12704491; doi:10.1515/nanoph-2025-0465)
Supplement: Supplementary file 1 — Supplementary Material Details [file j_nanoph-2025-0465_suppl_001.pdf]

Structured Beam-Driven Multipolar Mode Control in Nanoparticles: Supplementary Information

Asma Fallah[a]***, Eileen Otte***[a]

[a] *The Institute of Optics, University of Rochester, Rochester, New York 14627, USA*

This document includes supplementary information related to the main article 'Structured beam-driven multipolar mode control in nanoparticles' by A. Fallah and E. Otte. We provide details on the theoretical framework, namely, on the description of focused generalized cylindrical vector beams (FGCVBs), beam shape coefficients (Bromwich formulation), the Debye series expansion for determining Lorenz-Mie coefficients, as well as the calculation of the scattering cross section (SCS). Finally, we give additional insights into the impact of the numerical aperture (NA) on the SCS and into the far field radiation pattern.

1. Details on the Theoretical Framework

1.1. Focused Generalized Cylindrical Vector Beam

Figure 1 depicts our approach for structured beam-driven multipolar mode control in nanoparticles, also summarizing important parameters used in our theoretical framework. The incident focal field of a generalized cylindrical vector beam (GCVB) is a superposition of radially, azimuthally, and longitudinally polarized components:

$$\vec{E}(\rho,\varphi,z) = E_\rho\,\vec{e}_\rho + E_\varphi\,\vec{e}_\varphi + E_z\,\vec{e}_z, \tag{1}$$

where the E_r, E_φ, and E_z can be derived in O_{xyz} coordinate using the Richards-Wolf formalism [1, 2]; the electric field near the focus of a high-NA lens is:

$$E_\rho(\rho,\varphi,z) = A\cos\varphi_0 \int_0^{\theta'_{\max}} \cos^{1/2}(\theta')P(\theta')\sin\theta'\cos\theta' J_1(k\rho'\sin\theta')e^{ikz\cos\theta'}\,d\theta' \tag{2}$$

$$E_\varphi(\rho,\varphi,z) = A\sin\varphi_0 \int_0^{\theta'_{\max}} \cos^{1/2}(\theta')P(\theta')J_1(k\rho'\sin\theta')e^{ikz\cos\theta'}\,d\theta' \tag{3}$$

$$E_z(\rho,\varphi,z) = iA\cos\varphi_0 \int_0^{\theta'_{\max}} \cos^{1/2}(\theta')P(\theta')\sin^2\theta' J_0(k\rho'\sin\theta')e^{ikz\cos\theta'}\,d\theta' \tag{4}$$

with $\rho' = \sqrt{\rho^2+\rho_0^2-2\rho\rho_0\cos(\phi-\phi_0)}$, $\rho_0 = \sqrt{x_0^2+y_0^2}$ and $\phi_0 = \arctan(y_0/x_0)$ (as shown in Fig. 1). Here, $\theta'_{\max} = \arcsin(\mathrm{NA}/n_{\mathrm{medium}})$ is the maximal angle determined by the numerical aperture of the objective lens and $n_{\mathrm{medium}} = 1$. $P(\theta')$ is the pupil apodization function which in our analysis, $P_\theta = H\big(\sin\theta - 0.1\,\sin\theta_{\max}\big)$, and A is a normalization factor. Moreover, k and J_n are the wavenumber and the Bessel function of first kind with order n.

1.2. Beam Shape Coefficients: Bromwich Formulation

Classical Lorenz-Mie theory assumes plane wave illumination, where the incident field has a simple mathematical form. However, our tightly focused structured beam has a complex three-dimensional field distribution that varies significantly over the particle volume. We need a way to "translate" this complex incident field into a form that Lorenz-Mie theory can handle. The solution lies in expressing our incident field as a series expansion in the same spherical multipole basis functions used in Lorenz-Mie theory. The expansion coefficients are called beam shape coefficients. Moreover, we employ the Bromwich formulation [3] to solve the scattering problem. This approach allows us to express the electromagnetic fields in terms of two canonical solutions—transverse magnetic (TM) and transverse electric (TE) modes—while ensuring that the boundary conditions imposed by Maxwell's equations are satisfied. To calculate the beam shape coefficients, we use the integral localized approximation.

Email address: eotte@ur.rochester.edu (Eileen Otte)

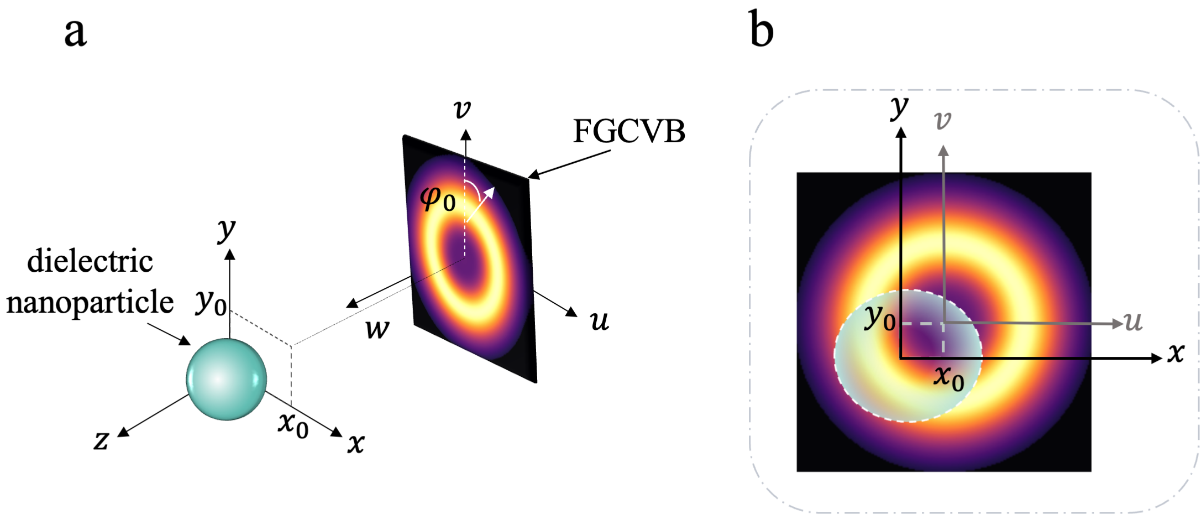

Figure 1: Schematic of the approach for multipolar mode control; (a) 3D and (b) front view. The dashed white circle in panel (b) highlights the boundary of the nanoparticle.

The key is that we can approximate the incident field locally (at the particle location) and integrate over the azimuthal direction [3, 4]:

$$g_{n,\mathrm{TE}}^{m} = \frac{Z_n^m}{2\pi H_0} \int_0^{2\pi} H_{r,\mathrm{loc}}(r,\theta,\phi) e^{-im\phi}\, d\phi \tag{5}$$

$$g_{n,\mathrm{TM}}^{m} = \frac{Z_n^m}{2\pi E_0} \int_0^{2\pi} E_{r,\mathrm{loc}}(r,\theta,\phi) e^{-im\phi}\, d\phi \tag{6}$$

where,

$$Z_n^m = \begin{cases} \dfrac{2n(n+1)i}{2n+1} & m = 0 \\ \left(\dfrac{-2i}{2n+1}\right)^{|m|-1} & m \neq 0 \end{cases} \tag{7}$$

The localized field components $E_{r,\mathrm{loc}}$ and $H_{r,\mathrm{loc}}$ represent the radial components of our structured incident field expressed in spherical coordinates centered on the particle. These components of the incident beam can be easily derived after transferring the beam into spherical coordinates, $E_r = E_\rho \sin\theta + E_z \cos\theta$ and considering $(\rho \to r\sin\theta, \phi \to \phi, z \to r\cos\theta)$, and inserting θ with $\pi/2$ and kr with $(n+1/2)$ [4, 5]. Therefore, the localized radial component of electric field can be derived as following:

$$E_{r,\mathrm{loc}}(\rho,\varphi,z) = A\cos\varphi_0 \int_0^{\theta'_{\max}} \cos^{3/2}(\theta') P(\theta') \sin\theta' J_1(\zeta^{1/2}\sin\theta')\, d\theta' \tag{8}$$

where $\zeta = (n+\frac{1}{2})^2 + \rho_0^2 k^2 - 2(n+\frac{1}{2})k\rho_0\cos(\phi-\phi_0)$. In addition, the radial component $H_{r,\mathrm{loc}}$ in spherical coordinates can be derived by utilizing the Maxwell relation as $H_\rho = \frac{1}{-i\omega}(\frac{1}{\rho}\partial E_z/\partial\phi - \partial E_\phi/\partial z)$ and the axial component H_z ($H_r = H_\rho \sin\theta + H_z\cos\theta$). Applying the localization approximation, we obtain:

$$H_{r,\mathrm{loc}} = \frac{A\cos\varphi_0 k^2\rho_0\sin(\phi-\phi_0)}{\omega\zeta^{1/2}} \int_0^{\theta'_{\max}} \cos^{1/2}(\theta') P(\theta') \sin^3\theta' J_1(\zeta^{1/2}\sin\theta')\, d\theta' + \frac{Ak}{\omega}\sin\varphi_0 \int_0^{\theta'_{\max}} \cos^{3/2}(\theta') P(\theta') J_1(\zeta^{1/2}\sin\theta')\, d\theta'. \tag{9}$$

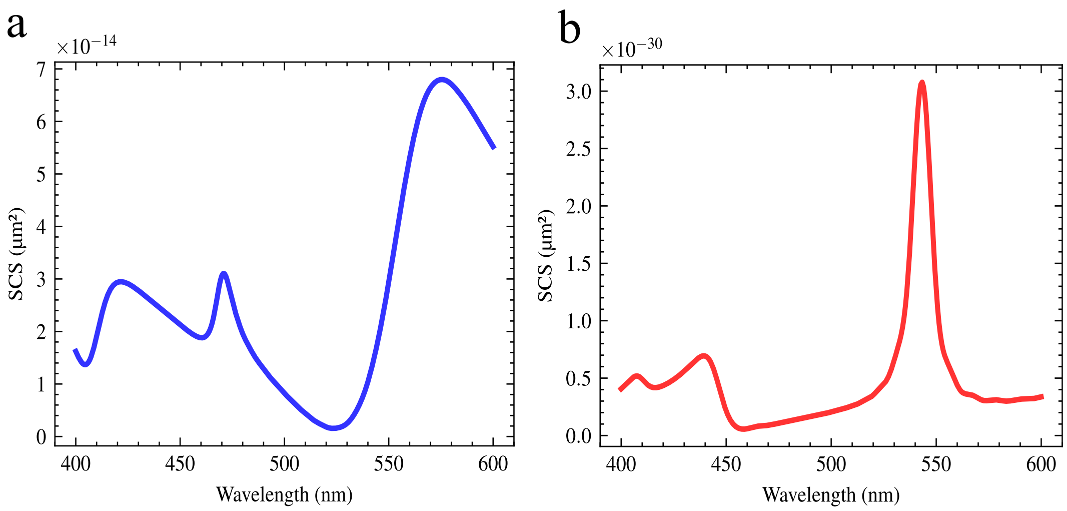

Figure 2: Non-normalized scattering cross section obtained from theoretical calculations for (a) a radially polarized incident beam ($\varphi_0 = 0$) and (b) an azimuthally polarized beam ($\varphi_0 = \pi/2$). The incident beam is focused through a lens with numerical aperture $NA = 0.8$, with $\rho_0 = 50$ nm and $\phi_0 = \pi/2$.

1.3. Debye Series

In order to gain physical insight into the scattering process, the Mie coefficients can be recast in terms of the Debye series expansion [6]. In this formulation, the total scattering is expressed as a superposition of distinct contributions corresponding to external reflection, transmission into the sphere, and multiple internal reflections before re-emergence. The relevant transmission and reflection coefficients are defined as following

$$T_n^{21} = \frac{m_1}{m_2}\frac{2i}{D_n}, \tag{10}$$

$$R_n^{212} = \frac{\alpha\,\xi_n^{(2)\prime}(\kappa_2)\,\xi_n^{(2)}(\kappa_1) - \beta\,\xi_n^{(2)}(\kappa_2)\,\xi_n^{(2)\prime}(\kappa_1)}{D_n}, \tag{11}$$

$$T_n^{12} = \frac{2i}{D_n}, \tag{12}$$

$$R_n^{121} = \frac{\alpha\,\xi_n^{(1)\prime}(\kappa_2)\,\xi_n^{(1)}(\kappa_1) - \beta\,\xi_n^{(1)}(\kappa_2)\,\xi_n^{(1)\prime}(\kappa_1)}{D_n}, \tag{13}$$

$$\tag{14}$$

with the common denominator

$$D_n = -\alpha\,\xi_n^{(1)\prime}(\kappa_2)\,\xi_n^{(2)}(\kappa_1) + \beta\,\xi_n^{(1)}(\kappa_2)\,\xi_n^{(2)\prime}(\kappa_1). \tag{15}$$

Here, $\kappa_j = m_j ka$ where k is free space wavenumber, a is the sphere radius, and m_j is the refractive index of medium j. The coefficients α and β depend on the polarization,

$$\alpha = \begin{cases} 1, & \text{for TE,} \\ \dfrac{m_1}{m_2}, & \text{for TM,} \end{cases} \qquad \beta = \begin{cases} \dfrac{m_1}{m_2}, & \text{for TE,} \\ 1, & \text{for TM.} \end{cases}$$

The spherical Ricatti–Hankel functions of first and second kinds are defined as

$$\xi_n^{(1)}(mkr) = mkr\,h_n^{(1)}(mkr), \tag{16}$$

$$\xi_n^{(2)}(mkr) = mkr\,h_n^{(2)}(mkr). \tag{17}$$

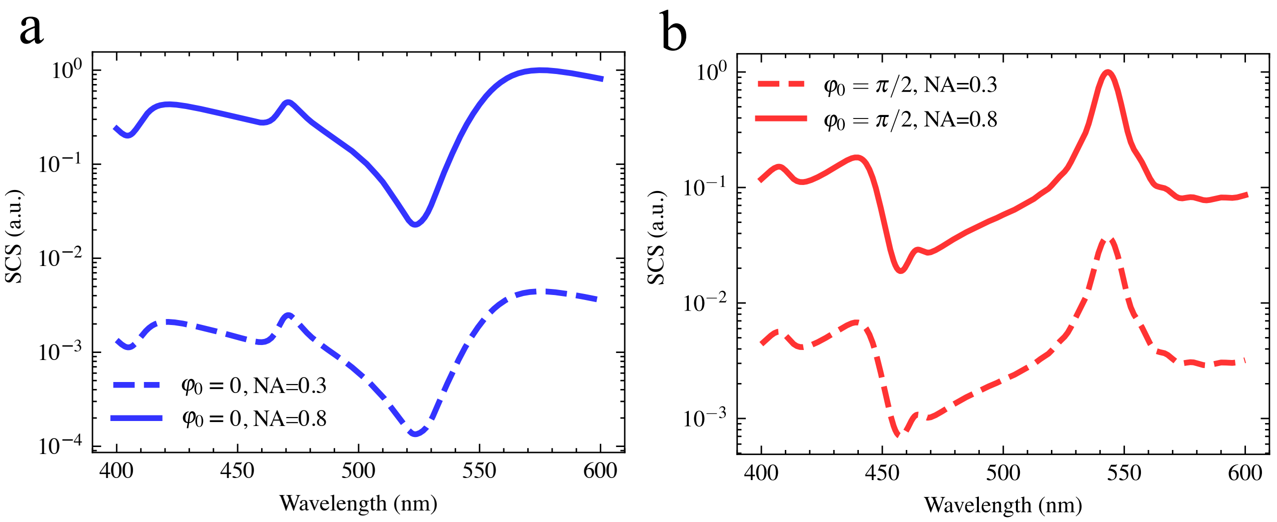

Figure 3: Scattering cross-section for (a) a radially polarized incident beam ($\varphi_0 = 0$) and (b) an azimuthally polarized beam ($\varphi_0 = \pi/2$), calculated for two numerical aperture values of the lens, NA $= 0.3$ (dashed) and NA $= 0.8$ (solid), with $\rho_0 = 50$ nm and $\phi_0 = \pi/2$.

with primes denoting differentiation with respect to the argument. Using these definitions, the Mie coefficients can be written as

$$\left\{ \begin{array}{c} a_n \\ b_n \end{array} \right\} = \tfrac{1}{2} \left[1 - R_n^{212} - \sum_{p=1}^{\infty} T_n^{21} \left(R_n^{121} \right)^{p-1} T_n^{12} \right]. \tag{18}$$

This representation separates the scattering into physically distinct terms. The first term corresponds to direct reflection at the sphere surface. The second term accounts for transmission into the external medium after internal reflection. The infinite series describes the contribution of waves that refract into the sphere, undergo $(p-1)$ internal reflections, and subsequently transmit out. In this way, the Debye expansion provides a clear link between the wave solution of Maxwell's equations and the ray-optics picture of scattering through external reflection, refraction, and multiple internal reflections. In practice, we truncate the internal-reflection sum at $p \le p_{max}$, in our analysis we use $p_{max} = 30$, which is sufficient for convergence in our parameter range.

1.4. Scattering Cross Section

The scattering cross section (SCS), which measures the particle's interaction strength with the incident beam, can be calculated by using Lorenz-Mie coefficients along with the beam shape coefficients as

$$C_{\text{sca}} = \frac{4\pi}{k^2} \sum_{n=1}^{n_{\max}} \sum_{m=-n}^{n} \left(\left| a_n g_{n,\text{TM}}^m \right|^2 + \left| b_n g_{n,\text{TE}}^m \right|^2 \right). \tag{19}$$

In our analysis $n_{\max}$ is chosen large enough to ensure convergence.

For completeness, Fig. 2 shows the non-normalized scattering cross section for the two orthogonal extreme cases of a focused (a) radial and (b) azimuthal GCVB.

1.5. Multipole Mode Decomposition

We partition the scattering into physically interpretable multipolar channels: electric dipole (ED, $n = 1$), magnetic dipole (MD, $n = 1$), electric quadrupole (EQ, $n = 2$), and magnetic quadrupole (MQ, $n = 2$). The incident field is expanded in vector spherical waves with beam–shape coefficients $g_{n,\text{TE}}^m$ and $g_{n,\text{TM}}^m$. For each (n, m), the scattered partial-wave amplitudes are

$$p_{nm}^E = a_n \, g_{n,\text{TM}}^m, \qquad p_{nm}^M = b_n \, g_{n,\text{TE}}^m,$$

where a_n and b_n are the Mie (Debye-series) coefficients. Channel amplitudes are formed by coherently summing the relevant partial waves,

$$A_n^E = \sum_{m=-n}^{n} p_{nm}^E, \qquad A_n^M = \sum_{m=-n}^{n} p_{nm}^M,$$

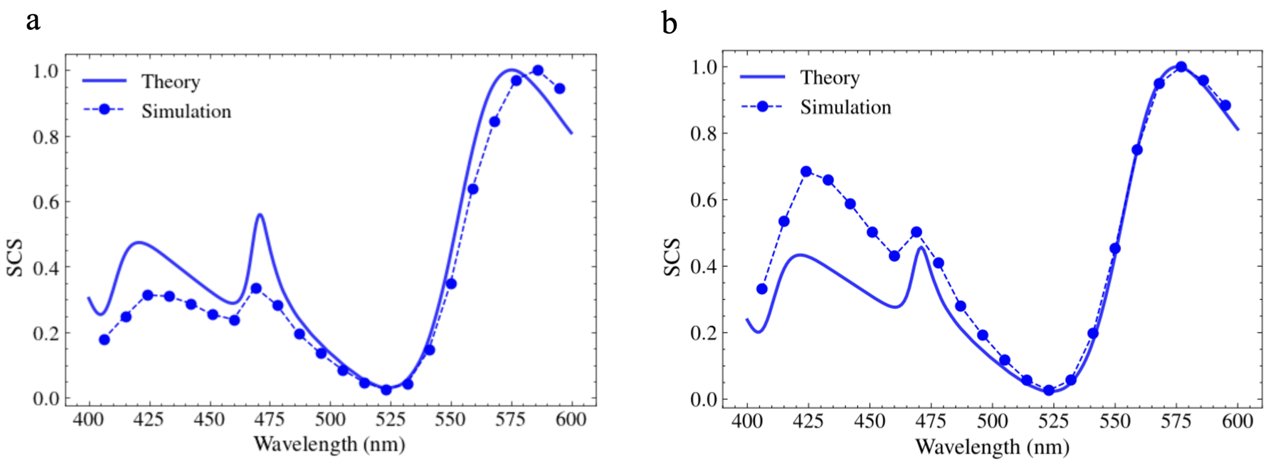

Figure 4: Scattering cross section obtained from theoretical calculations and FDTD simulations for a radially polarized incident beam, for (a) NA=0.3 and (b) NA=0.8.

and grouped as

$$\text{Dipole: } (A_{\mathrm{D}}^{E}, A_{\mathrm{D}}^{M}) = (A_{1}^{E}, A_{1}^{M}), \quad \text{Quadrupole: } (A_{\mathrm{Q}}^{E}, A_{\mathrm{Q}}^{M}) = (A_{2}^{E}, A_{2}^{M}),$$

The channel-resolved scattering cross sections are then

$$C_{\mathrm{D}}^{E} = \frac{4\pi}{k^2}|A_{\mathrm{D}}^{E}|^2, \quad C_{\mathrm{D}}^{M} = \frac{4\pi}{k^2}|A_{\mathrm{D}}^{M}|^2,$$

$$C_{\mathrm{Q}}^{E} = \frac{4\pi}{k^2}|A_{\mathrm{Q}}^{E}|^2, \quad C_{\mathrm{Q}}^{M} = \frac{4\pi}{k^2}|A_{\mathrm{Q}}^{M}|^2.$$

2. The Impact of the Numerical Aperture of the Lens on the SCS

Figure 3 depicts the influence of the numerical aperture of the lens on the SCS for two polarizations: (a) radial and (b) azimuthal, in the case of an off-axis beam. The numerical aperture primarily affects the magnitude of the SCS rather than its spectral behavior. A comparison of panels (a) and (b) shows that, for lower numerical apertures, the SCS is reduced by approximately two orders of magnitude for radial polarization and by one order for azimuthal polarization. The weak impact of the numerical aperture can be explained by the size ratio of particle and illuminating beam: the particles nanoscale size let it only experience a small central part of the beam, which changes less significantly with NA. Additionally, note that the accuracy of theoretical analysis depends on the numerical aperture of the lens, as illustrated in Fig. 4.

3. Far Field Radiation Pattern of the Nanoparticle

Fig. 5 compare the angular scattering distributions of radial and azimuthal polarization at $\lambda = 575$ nm. Here, in spherical coordinates, at each angle θ, we take the intensity at all ϕ angles and average them. This gives us a 2D cross-section of the full 3D radiation pattern. It is clear that, electric dipole modes (radial) scatter about 10 more strongly than magnetic dipoles (azimuthal) at this wavelength. Furthermore, in both cases, the backward hemisphere ($\theta > 90°$) contains significantly more scattered power than the forward hemisphere ($\theta < 90°$), resulting in a forward-to-backward ratio $F/B < 1$.

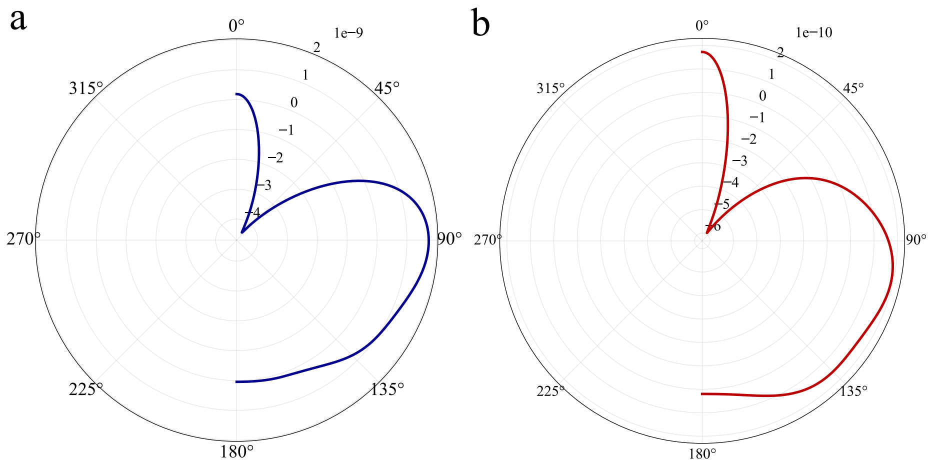

Figure 5: Comparison of angular scattering distributions for (a) radial and (b) azimuthal polarization at $\lambda = 575$ nm. Each graph presents the intensity dependent on θ with each data point corresponding to the average of intensity at all ϕ angles (spherical coordinates). Here, the incident beam parameter are NA $= 0.8$, $\rho_0 = 50$ nm and $\phi_0 = \pi/2$. Backwards/ forwards scattering: $\theta > 90°$/ $\theta < 90°$.

References

[1] K. S. Youngworth, T. G. Brown, Focusing of high numerical aperture cylindrical-vector beams, Optics Express 7 (2) (2000) 77–87.

[2] Q. Zhan, J. R. Leger, Focus shaping using cylindrical vector beams, Optics Express 10 (7) (2002) 324–331.

[3] G. Gouesbet, G. Gréhan, B. Maheu, Scattering of a gaussian beam by a mie scatter center using a bromwich formalism, Journal of Optics 16 (2) (1985) 83.

[4] L. A. Ambrosio, H. E. Hernández-Figueroa, Integral localized approximation description of ordinary bessel beams and application to optical trapping forces, Biomedical Optics Express 2 (7) (2011) 1893–1906.

[5] K. F. Ren, G. Gouesbet, G. Gréhan, Integral localized approximation in generalized lorenz–mie theory, Applied Optics 37 (19) (1998) 4218–4225.

[6] R. Li, X. Han, H. Jiang, K. F. Ren, Debye series for light scattering by a multilayered sphere, Applied Optics 45 (6) (2006) 1260–1270.
